# The adenosine A2b receptor promotes tumor progression of bladder urothelial carcinoma by enhancing MAPK signaling pathway

**DOI:** 10.18632/oncotarget.17835

**Published:** 2017-05-12

**Authors:** Yihong Zhou, Xi Chu, Fei Deng, Liang Tong, Guoxiong Tong, Ye Yi, Jianye Liu, Jin Tang, Yuxin Tang, Yang Xia, Yingbo Dai

**Affiliations:** ^1^ Department of Urology, The Third Xiangya Hospital of Central South University, Changsha, China; ^2^ Department of Biochemistry and Molecular Biology, University of Texas Medical School, Houston, Texas, USA

**Keywords:** adenosine A2b receptor, bladder urothelial carcinoma, prognosis, MAPK signaling

## Abstract

The adenosine A2b receptor (A2bR) was considered to play an oncogenic role in many human malignancies. However, the expression and molecular function of A2bR in bladder urothelial carcinoma (BUC) have not been well elucidated. Herein, we found that the expression of A2bR was higher than other adenosine receptors in BUC tissues and cells, and it was upregulated in BUC tissues compared with matched normal bladder tissues. Furthermore, high expression of A2bR was associated with poor prognosis of patients with BUC. In addition, suppression of A2bR inhibited the proliferation, migration and invasion of BUC cells and arrested the cell cycle at the G1 phase. Finally, we demonstrated that downregulation of A2bR inhibited the proliferation, migration and invasion of BUC in part via the MAPK signaling pathway, increasing the levels of P21 but decreasing the levels of cyclin B1, D, E1, MMP-2 and MMP-9. Overexpression of MMP-2 could rescue BUC cells migration and invasion. Thus, the present study indicates that A2bR may play a potential oncogenic role in BUC progression and act as a potential biomarker to identify BUC patients with poor clinical outcomes.

## INTRODUCTION

Bladder urothelial carcinoma (BUC) is the second most common genitourinary malignancy leading causes of cancer-related death in western countries [1]. Clinically, surgical treatment is the main method for patients with non-muscle invasive bladder cancer (NMIBC) or muscle-invasive bladder cancer (MIBC). However, despite advances in surgical technique, the long-term prognosis of BUC patients after treatment is still poor. It has been reported about 1/3 of NMIBC patients would relapse and progress, and the 5-year cancer-specific survival is just only 50-60% for MIBC [2–4]. Moreover, the therapeutic response for patients with advanced BUC to radiotherapy or chemotherapy remains unsatisfactory. Thus, it is important to understand the underlying molecular mechanisms and identify new promising biomarkers that can be used to define the progressive and metastatic potential.

Adenosine is an endogenous purine nucleoside and generates from the degradation of ATP. In our previous studies, we found adenosine concentration elevated under the inflammatory, ischemic or hypoxia conditions [5–7]. Adenosine acts its biological functions via four subtypes of adenosine receptors (ARs), A1, A2a, A2b, and A3 [8]. Recently, studies indicated that A2b receptor (A2bR) was highly expressed in various tumors due to the hypoxic environments of solid tumors [9, 10]. Suppression of A2bR expression with shRNA was observed to significantly inhibit cell proliferation in oral cancer [10]. Moreover, the A2bR antagonist could slow the growth of MB49 bladder and 4T1 breast tumors [11]. These findings, collectively, suggest that A2bR has a potentially oncogenic function. However, the expression pattern and biological function of A2bR in BUC have not been elucidated. In the present study, the expression of A2bR in both BUC tissues and cell lines was conducted. The potential role and its underlying molecular mechanisms in BUC were further investigated.

## RESULTS

### A2bR was highly expressed in BUC

**Figure 1:**
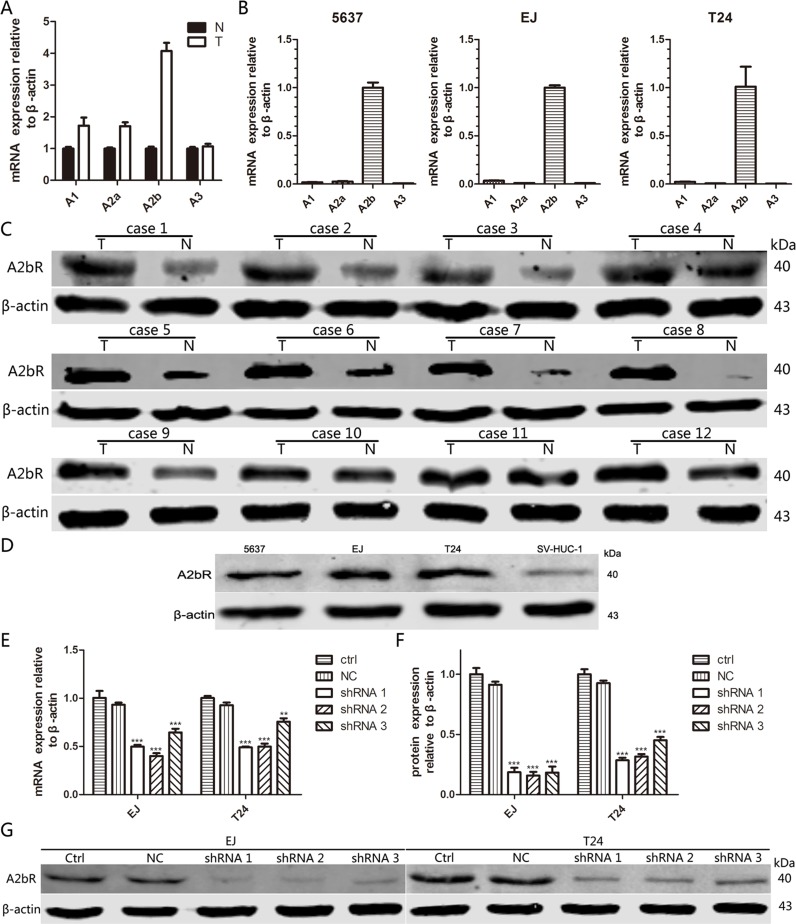
A2bR is the main subtype of ARs and upregulated in BUC **A**. mRNA expression of four subtypes of ARs in BUC tissues (T) and adjacent normal tissues (N). **B**. mRNA expression of four subtypes of ARs in three BUC cell lines. **C**. Protein expression of A2bR in 12 pairs of fresh BUC tissues and their matched normal urothelial bladder epithelial tissues. **D**. Protein expression of A2bR in three BUC cell lines and normal human urinary tract epithelial cell line. **E**., **F**. and **G**. qRT-PCR and western blot analyses of knockdown efficiency with shRNA transfection in EJ and T24 cells. The graph shows the mean ± SD; ***P < 0.001, **P < 0.01.

To identify the expression of different subtypes of ARs, we examined ARs expression at mRNA as well as protein level in three bladder cancer cell lines, the normal human urinary tract epithelial cell line (SV-HUC-1), 12 pairs of fresh BUC tissues and matched normal urothelial bladder epithelial tissues. As shown in Figure 1A and 1B, the expression of A2bR was higher than other three adenosine receptors in BUC tissues and all three BUC cell lines. Moreover, western blotting assay showed that A2bR was highly expressed in these fresh BUC tissues compared to the matched normal urothelial bladder epithelial tissues (Figure 1C). Compared with SV-HUC-1, A2bR expression was significantly increased in all three BUC cell lines, especially in the EJ and T24 lines (Figure 1D).

### A2bR expression was correlated with poor prognosis in BUC patients

**Figure 2:**
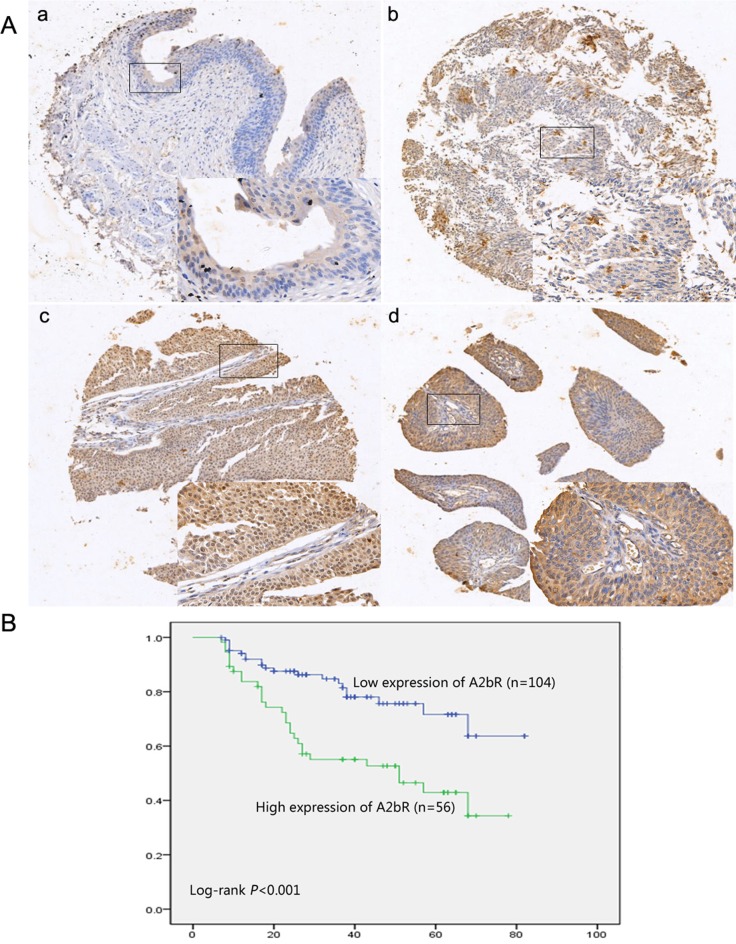
High expression of A2bR in BUC is associated with poorer prognosis **A**. Representative images of A2bR expression in normal urothelial bladder epithelial tissue and BUC specimens examined by IHC (200×). The normal urothelial bladder epithelial tissue was stained negatively (a), and the BUC tissues showed low (b) and high (c and d) expression of A2bR. **B**. Kaplan-Meier analysis of survival in patients between low expression of A2bR group (*n* = 104) and high expression of A2bR group (*n* = 56).

Among the BUC tissues, 65% (104/160) of the cases showed low expression of A2bR, whereas among the remaining samples 35% (56/160) had high staining for A2bR (Figure 2A). No significant association was found between the level of A2bR expression and age, gender, tumor grade, T stage or tumor multiplicity (Table 1). Kaplan-Meier survival analysis and the log-rank test showed that A2bR expression was markedly associated with poor prognosis in BUC patients (Figure 2B). Patients with low A2bR expression had better overall survival. Cox regression analyses revealed that in this study, A2bR expression was an independent prognostic marker (Table 2). Thus, our study indicated that the A2bR expression level had a negative correlation with prognosis in BUC patients.

**Table 1:** The relationship between A2bR expression and clinicopathological features of BUC patients

Parameters	Total cases	A2bR expression		*X*^2^	*P* value
		Low expression	High expression		
Age (years)				2.261	0.133
≥60	90	63	27		
<60	70	41	29		
Gender				0.724	0.395
Male	126	84	42		
Female	34	20	14		
Tumor grade				0.288	0.592
1	50	34	16		
2-3	110	70	40		
T stage				3.355	0.067
1	51	28	23		
2-3	109	76	33		
Tumor multiplicity				0.271	0.603
Unifocal	71	42	29		
Multifocal	89	49	40		

**Table 2:** Univariate and multivariate analyses of clinicopathological parameters and A2bR for overall survival in patients with BUC

Variables	Univariate analysis		Multivariate analysis	
	HR (95% CI)	*P* value	HR (95% CI)	*P* value
Age (<60 *vs*. ≥60 years)	1.103 (0.988-1.039)	0.303		
Gender (Male *vs*. Female)	0.629 (0.295-1.343)	0.231		
Tumor grade (G1 *vs*. G2-G3)	1.554 (0.777-3.108)	0.213		
T stage (T1 *vs*. T2-T3)	0.706 (0.399-1.248)	0.231		
Tumor multiplicity (Unifocal *vs*. Multifocal)	2.227 (1.267-3.913)	0.005	1.722 (0.946-3.135)	0.075
A2bR expression (Low *vs*. High)	2.605 (1.485-4.570)	0.001	2.173 (1.198-3.943)	0.001

### A2bR knockdown inhibited the proliferation and arrested human BUC cell lines at the G0/G1 phase

**Figure 3:**
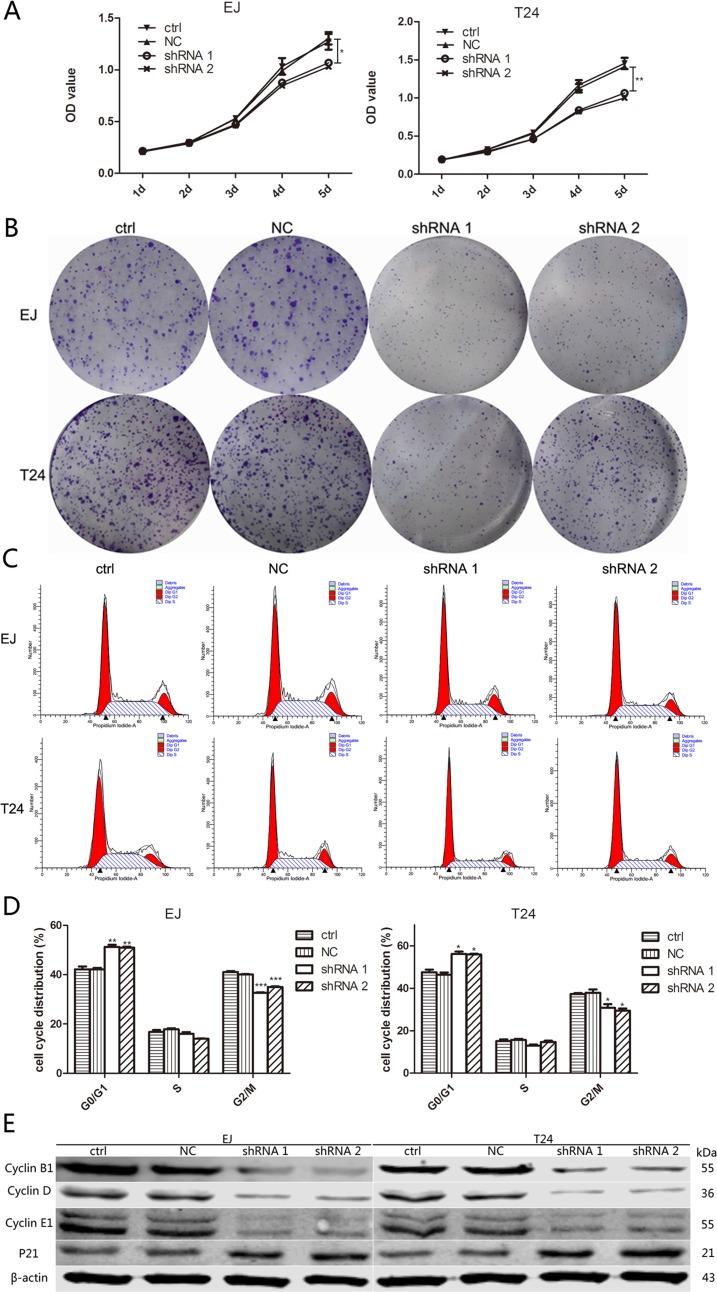
Downregulation of A2bR inhibits cell growth of BUC cells in vitro, leads to a G0/G1 phase cell cycle arrest and reduces the level of cyclin family and P21 **A**. and **B**. Suppression of A2bR inhibits cell growth as detected by MTT (A) and colony formation assays (B). **C**. and **D**. Flow cytometry analysis shows that knockdown of A2bR expression increases the percentage of cells in G0/G1 phase and decreases the percentage of cells in G2/M phase. **E**. Downregulation of A2bR upregulates P21 but downregulates cyclin B1, D and E1. The graph shows the mean ± SD; ***P < 0.001, **P < 0.01, *P < 0.05.

To explore the impact of A2bR on BUC cells proliferation, short hairpin RNAs (shRNA 1, shRNA 2 and shRNA 3) were used to knock down A2bR expression in EJ and T24 cells. Western blot and qRT-PCR analysis were performed after transfection, and the results showed stable A2bR knockdown cell lines were made (Figure 1E and 2F). MTT assay showed that the downregulation of A2bR expression decreased the proliferation of EJ and T24 cells as compared with the control cells (Figure 3A; *P* < 0.05). Similarly, colony formation capacity was reduced following A2bR knockdown (Figure 3B; *P* < 0.05).

We next studied the effects of A2bR knockdown on cell cycle progression by flow cytometry analysis. The results showed reduced expression of A2bR was associated with G0/G1 cell cycle arrest. The proportion of G0/G1 phase cells was significantly increased after A2bR downregulation in EJ (41.3±1.5 versus 51.9±1.3 versus 51.4±0.9, NC versus shRNA1 versus shRNA2, respectively) and T24 (45.7±1.6 versus 56.9±1.7 versus 56.4±0.9, NC versus shRNA1 versus shRNA2, respectively) cells (Figure 3C and 3D). To further explore the mechanism underlying the inhibitory effect of A2bR knockdown on cell growth, we analyzed the protein expression of cyclin family and P21. As shown in Figure 3E, downregulation of A2bR expression decreased the levels of cyclin B1, D and E1 but increased the level of P21. These data suggested that A2bR might be correlated with the proliferation potentialities of BUC cells by regulating the cell cycle.

### A2bR knockdown inhibited the migration and invasion of BUC cells by decreasing MMP-2 and MMP-9

**Figure 4:**
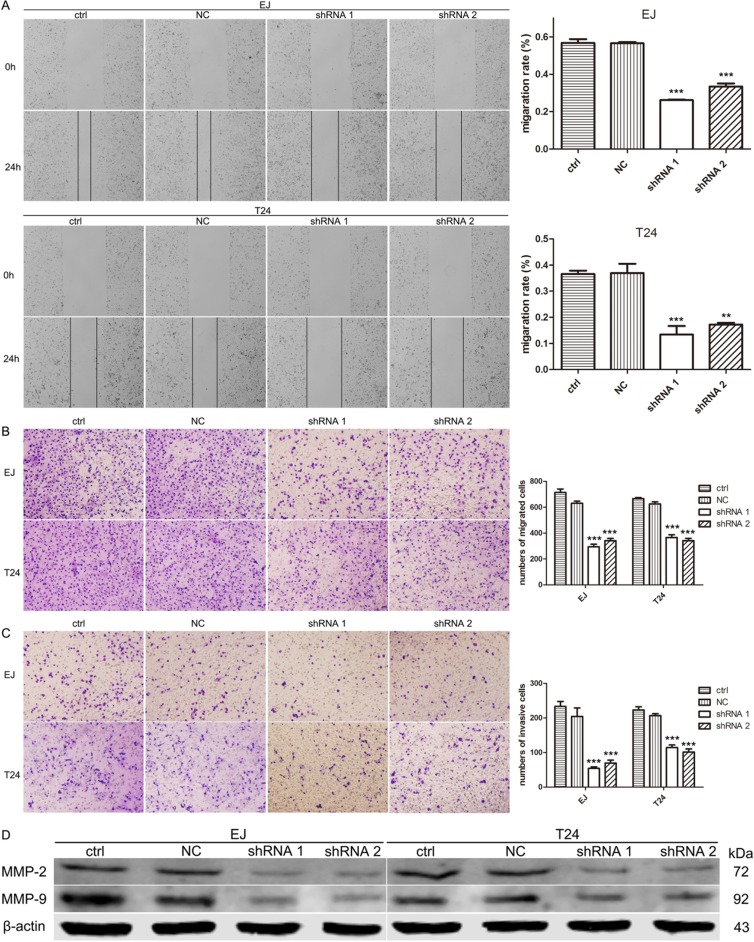
Downregulation of A2bR inhibits cell migration and invasion of BUC cells in vitro and reduces the level of MMP-2 and MMP-9 **A**. Wound healing assay shows EJ and T24 cells with A2bR knockdown migrate into the scratching area more slowly than control cells. **B**. and **C**. Suppression of A2bR reduces EJ and T24 cells migratory (B) and invasive (C) ability in a transwell assay without/with Matrigel. (D) Suppression of A2bR downregulates MMP-2 and MMP-9. The graph shows the mean ± SD; ***P < 0.001, **P < 0.01.

To examine the potential role of A2bR on the migration and invasion of BUC cells, wound healing assay and transwell migration and invasion assay were performed. The results of wound healing assay showed that BUC cells with A2bR knockdown migrated into the scratching area more slowly than control cells (Figure 4A, *P* < 0.001). To further investigate these observations, transwell assays without/with Matrigel both indicated that downregulation of A2bR expression decreased the migration and invasion capability compared with control cells (Figure 4B and 4C, P < 0.001). These results suggested that A2bR contributed to the migratory and invasive abilities of BUC cells.

**Figure 5:**
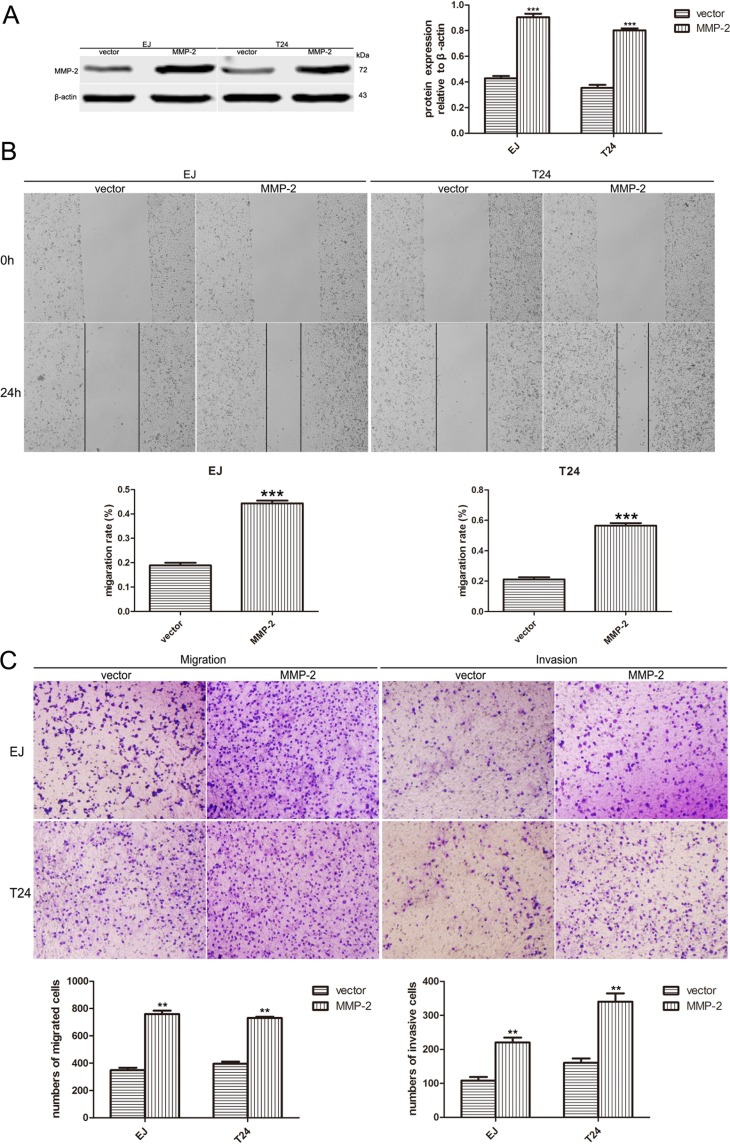
Overexpression of MMP-2 rescues cell migration and invasion of BUC cells *in vitro* **A**. After A2bR knockdown cells are stable constructed, MMP-2 is overexpressed in EJ and T24 cells detected by western blot analyses. **B**. Wound healing assay shows EJ and T24 cells with MMP-2 overexpression migrate into the scratching area faster than control cells. **C**. MMP-2 overexpression reduces EJ and T24 cells migratory and invasive ability in a transwell assay without/with Matrigel. The graph shows the mean ± SD; ***P < 0.001, **P < 0.01.

We next examined the expression of MMP-2 and MMP-9, which are widely known as critical molecules involved in cancer invasion and metastasis. As shown in Figure 4D, reducing the level of A2bR could downregulate MMP-2 and MMP-9. To explore whether MMP-2 or MMP-9 participates in A2bR mediated promotion of BUC cell migration and invasion, MMP-2 overexpression was performed using the pcDNA3.1-MMP-2 after the stable A2bR knockdown cells were constructed (Figure 5A, *P* < 0.001). We found that overexpression of MMP-2 could rescue BUC cells migration and invasion (Figure 5B and 5C). Together, these results indicated that A2bR knockdown inhibited the migration and invasion of BUC cells by decreasing MMP-2 and MMP-9.

### A2bR knockdown inhibited tumor growth in nude mice

**Figure 6:**
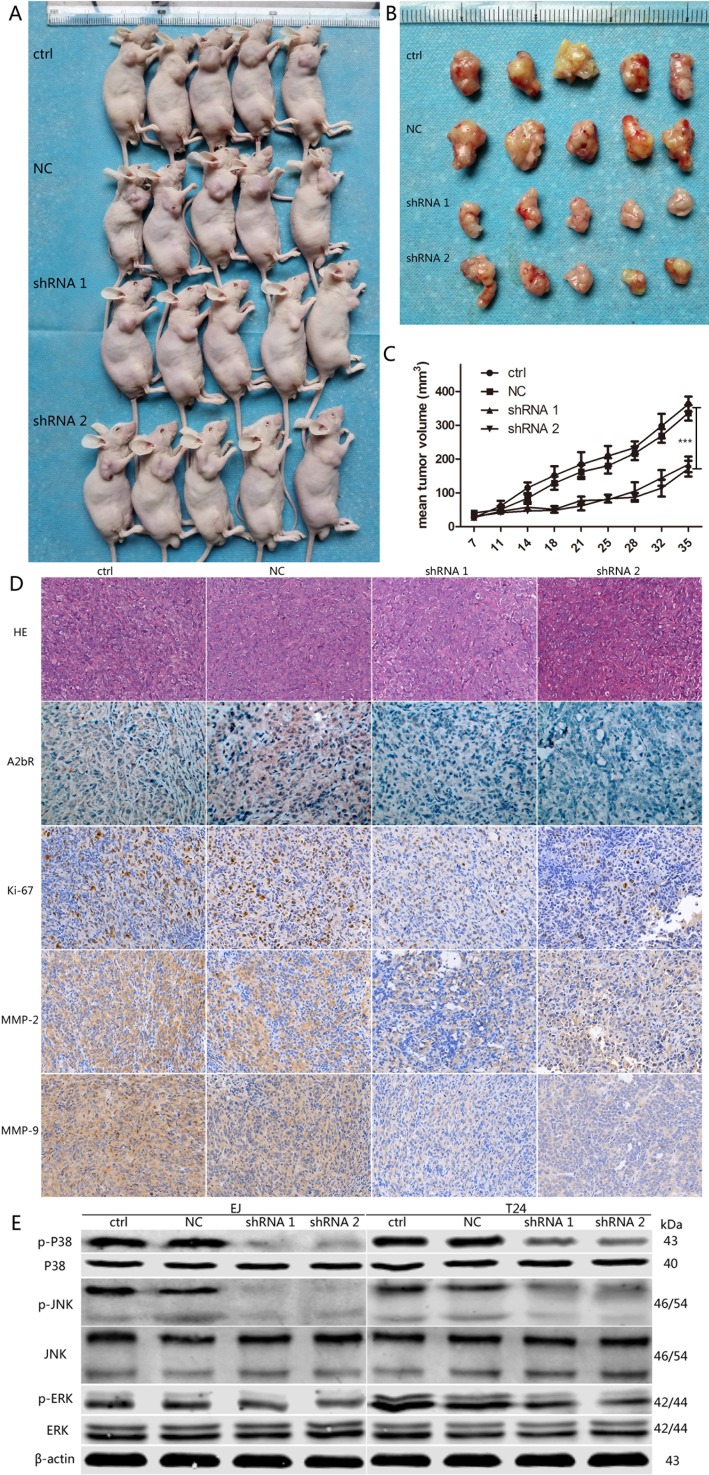
Downregulation of A2bR inhibits tumor growth of T24 cells *in vivo*, and regulates expression of MAPK pathway proteins in EJ and T24 cells **A**. and **B**. Tumors were removed at the 35th day after nude mice received subcutaneous injection (*n* = 5 for each group). **C**. Tumor growth curve. **D**. HE staining (200×) and IHC (200×) for A2bR, Ki-67, MMP-2 and MMP-9 in BUC xenografts. **E**. Suppression of A2bR decreases the phosphorylation levels of P38, JNK and ERK. The graph shows the mean ± SD; ***P < 0.001.

As described above, we discovered that A2bR knockdown could restrain the growth rate of BUC cells *in vitro*. We next assessed the effect of A2bR on the tumor formation *in vivo*. At the 35th day after nude mice received subcutaneous injection, tumors were completely removed (Figure 6A and 6B). We found the average tumor volume decreased in the group inoculated with A2bR knockdown cells (184.4±51.3 and 171.8±51.6, shRNA1 and shRNA2, respectively), compared with the control groups (362.8±49.5 and 336.8±51.4, ctrl and NC, respectively) (Figure 6C). Moreover, IHC also showed areas with low levels of A2bR exhibited low Ki-67, MMP-2 and MMP-9 in the tumors inoculated with A2bR knockdown cells (Figure 6D). These data showed that A2bR knockdown could decrease the ability of BUC cells to form tumor in nude mice.

### A2bR knockdown inhibited malignancy of BUC cells through the MAPK pathway

To explore the mechanism by which A2bR influences tumor growth, migration and invasion in BUC cells, we examined the expression of relevant proteins in the MAPK pathway. We observed that downregulation of A2bR dramatically decreased the levels of phosphorylated P38 (p-P38), phosphorylated JNK (p-JNK) and phosphorylated ERK (p-ERK) (Figure 6E). These results suggested that A2bR knockdown inhibited malignancy of BUC cells, at least partly, through the MAPK pathway.

## DISCUSSION

The adenosine A2b receptor is the subtype which binds to adenosine with lowest affinity, therefore it is considered to play an important role in different diseases [12]. During the last decade, more and more studies focused on the relationship between A2bR and cancer. It has been documented that the expression level of A2bR was the highest among the four adenosine receptor subtypes in different ovarian and prostate cancer cell lines [9, 13]. Similarly, compared to the adjacent normal tissues, the mean expression of A2bR was increased in diverse human cancers, including ovarian, lung, liver, oral, colon and prostate cancers [9, 10, 14–17]. In addition, high A2bR expression in triple negative breast cancer was significantly associated with poor prognosis [18]. Using the Oncomine database (https://www.oncomine.org), similar results were also observed in other human malignancies, such as colorectal, renal and prostate cancers, in which patients with high expression of A2bR always had a worse overall survival [19]. However, the association between A2bR and prognosis of BUC has not been reported to date. In the present study, our results showed that the expression of A2bR was higher than other ARs in BUC tissues and cells. When compared with their matched normal bladder tissues, the mRNA and protein levels of A2bR were upregulated in BUC tissues. High expression of A2bR in our BUC cohorts was positively associated with poor prognosis of patients. Theses findings suggest that upregulation of A2bR might act as a potential biomarker to identify BUC patients with poorer clinical outcomes.

Upon activation, A2bR can link to the Gs and Gq protein stimulating cAMP synthesis and triggering the activation of phospholipase C [20, 21]. It was reported that A2bR was involved in several important mechanisms, such as regulating cell proliferation. Using the A2bR antagonist, MRS1754 or PBS603 or ATL801, the growth of cancer cells was inhibited *in vitro* and *in vivo* [11, 17, 22]. Similar results were also observed by suppressing the expression of A2bR with shRNA in human oral squamous cell carcinoma derived cell lines [10]. In contrast, under extracellular adenosine stimulation, upregulation of A2bR could increase the cell growth [23]. In the present study, we found the protein level of A2bR in two human BUC cell lines EJ and T24 was higher than that of the normal human urinary tract epithelial cell line (SV-HUC-1). Further functional studies indicated that the suppression of A2bR expression by transfection with shRNA in both EJ and T24 cells led to reducing cell growth *in vitro* and inhibiting tumorigenicity *in vivo*. Notably, A2bR knockdown arrested human BUC cell lines at the G0/G1 phase, but was not associated with cell apoptosis (Supplementary Figure S1). Moreover, to further explore the mechanism underlying the inhibitory effect of A2bR knockdown on cell cycle, the protein levels of cyclin family and P21 were examined. Our results showed that suppression of A2bR could upregulate P21 but downregulate cyclin B1, D and E1. In the light of these findings, we thought that A2bR might link to the cell cycle regulation.

Several studies have demonstrated that high expression of A2bR was positively correlated with T classification in oral [10], lung [15] and liver cancer [16], suggesting that A2bR expression may be associated with cell invasion and/or metastasis. Interestingly, the experimental and spontaneous metastasis could be decreased by A2bR antagonist or knockdown of A2bR in mouse models of melanoma and triple-negative breast cancer [18]. Knockdown of A2bR in LM2 cells could also limit metastasis in mouse models and suppress cell migration *in vitro* [24]. In our study, cell migration and invasion ability were significantly reduced by downregulation of A2bR *in vitro*. In addition, MMP-2 and MMP-9 have been linked to tumor cells migratory and invasive ability [25, 26]. Our study demonstrated that suppression of A2bR expression could decrease the level of MMP-2 and MMP-9, and overexpression of MMP-2 rescued the migratory and invasive capacity of BUC cells. In this regard, we considered A2bR might be involved in the migratory and invasive process via modulating the expression of MMP-2 and MMP-9.

**Figure 7:**
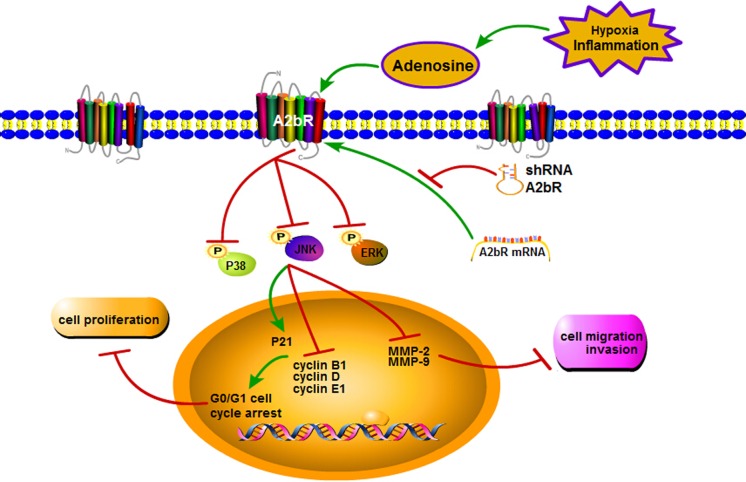
An effect and mechanism model that A2bR knockdown inhibits tumor growth and invasion through MAPK signaling pathway in BUC Suppression of A2bR expression reduces the phosphorylation levels of P38, JNK and ERK. Moreover, it upregulates P21 but downregulates cyclin B1, D, E1, MMP-2 and MMP-9, which leads to inhibition of BUC cells proliferation, migration and invasion.

To date, however, the underlying molecular mechanisms by which A2bR regulates tumor proliferation and migration/invasion remain poorly understood. It was reported that A2bR was correlated with FOS-related antigen 1 (FRA1) in the progress of breast cancer [24]. Interestingly, FRA1 was found to interact with the MAPK signaling pathway [27], which acted a key role in cell proliferation, migration and invasion. Importantly, suppression of A2bR expression with shRNA could decrease the phosphorylation level of ERK in oral cancer cells [10] and the A2bR antagonist could reduce P38 and ERK phosphorylation basal levels in A375 cells [28]. Therefore, we further observed the related proteins expression of MAPK signaling pathway. Our results showed that knocking down the expression of A2bR reduced the phosphorylation level of P38, JNK and ERK. These observations, together with the results of A2bR functional studies in the BUC cells, do suggest that the downregulation of A2bR expression may inhibit the cell proliferative, migratory and invasive ability through the MAPK signaling pathway (Figure 7).

In summary, results from the present study, for the first time, show a high expression of A2bR in BUC tissues and cell lines. Our study indicates that high expression of A2bR may be a valuable prognostic marker of BUC progression. Downregulation of A2bR plays a key role in inhibiting cell proliferation, migration and invasion via the MAPK signaling pathway. Suppressing A2bR expression may, at least in part, be a potentially effective treatment for BUC.

## MATERIALS AND METHODS

### Patients and specimens

The study included a total of 160 bladder urothelial carcinoma patients who were histologically diagnosed at the Third Xiangya Hospital of Central South University between 2008 and 2013. All the BUC specimens were soon embedded in paraffin after resection. In addition, 12 fresh pairs of BUC tissues and the matched normal bladder mucosa tissues (>3 cm from the margin of tumor) were immediately stored at −80°C for further use. For the research purposes, prior patient's consent and approval from the ethics committee of Central South University were obtained.

### Cell cultures and transfection

The normal human urinary tract epithelial cell line (SV-HUC-1) and human bladder cancer cell lines (5637, EJ, T24) were generously provided by Zhou F (State Key Laboratory of Oncology in Sun Yat-sen University). SV-HUC-1 was cultured in F-12K medium (Hyclone) and 5637, EJ, T24 were cultured in RPMI 1640 medium (Hyclone) with 10% fetal bovine serum (Hyclone).

The MMP-2 expression vector and empty vector were synthesized and constructed by Shanghai GenePharma Co. The pcDNA3.1-MMP-2 or negative control was transfected into cells using Lipofectamine 3000 reagent (Invitrogen, Carlsbad, USA) according to the manufacturer's protocol.

### Immunohistochemistry (IHC) analysis

A2bR (1:80 dilution, Abcam, cat #40002, USA) was applied. Bladder cancer tissue was stained according to procedures previously described [6]. Scoring for IHC staining was carried out based on the staining intensity and the area of positive cells. The staining intensity was divided as 0 (negative), 1 (weak), 2 (medium) and 3 (intense). The area of positive cells was scored as 0 (0%), 1 (1-25%), 2 (26-50%), 3 (51% -75%) or 4 (76-100%). The final staining scores were calculated by multiplying the above two scores, ranging from 0 to 12. For the purpose of statistical evaluation, final staining scores of 0-4 and 5-12 were respectively considered to be low and high expressions. The staining results were assessed and confirmed by two independent pathologists blinded to the clinical data.

### Lentivirus-mediated A2bR RNA interference

Lentivirus encoding shA2bR were designed and purchased from Shanghai GenePharma Co. Three short hairpin RNA oligonucleotides (shRNA 1: 5′-GAGCTCCATCTTCAGCCTTCT-3′; shRNA 2: 5′-GGCATCGGATTGACTCCATTC-3′; shRNA 3: 5′-GCCACCAACAACTGCACAGAA-3′) and a nonsense shRNA (NC: 5′-TTCTCCGAACGTGTCACGT-3′) were designed. BUC cells that were not transfected with shRNA were used as control group (ctrl). Stable transfectants were selected with puromycin (2 mg/ml) and verified by quantitative real-time PCR analysis and western blotting.

### Quantitative real-time PCR

Total RNA was extracted from tissues or cells using TRIzol reagent (Invitrogen, Carlsbad, CA, USA) according to the manufacturer's protocol. Quantitative real-time PCR was performed using the SYBR Green PCR kit and β-actin was used as the internal control. The sequences of the primers were shown in Supplementary Table S1.

### Western blotting

Cell lysates or tissues were extracted by RIPA buffer. The proteins concentrations were detected by using a bicinchoninic acid (BCA) assay (Beyotime, Shanghai, China). Next the proteins were separated in 10% SDS-polyacrylamide gel electrophoresis (PAGE) and transferred on polyvinylidend difluoride membranes. The membranes were then blocked and incubated with primary antibodies. After incubation with a secondary antibody, the immunocomplexs were visualized by enhanced chemiluminescence (SuperSignal Pierce Biotechnology). β-actin was used as the loading control. The following antibodies against P38 (#8690), p-P38 (#4515), ERK (#4695), p-ERK (#4370), JNK (9252), p-JNK (#4668), MMP-2 (#13132), MMP-9 (#13667) and β-actin (#12620) were purchased from Cell Signaling technology. Antibodies against P21 (60214-1-Ig), cyclin D (60186-1-Ig), cyclin E1 (11554-1-AP), cyclin B1 (55004-1-AP) were obtained from Proteinch.

### MTT assay and colony formation assay

The mock or shRNAs transfected cells were seeded onto 96-well plates at a density of 2000 cells per well. During a 5-d culture periods, 20μl MTT-medium mixed solution (0.5 mg/ml) was added to cell culture per day, and incubated for 4h. The MTT solution was then discarded and 150μl dimethyl sulfoxide (DMSO) was added to dissolve the formazan sediment. The optical density was assayed at 490 nm using a microplate absorbance reader.

For colony formation assay, the cells were seeded in 6-well culture plate with 800 cells per well and cultured for 10-12d. Then the cells were stained with Crystal violet.

### Wound healing assay

After transfected with shRNA, EJ and T24 cells were plated into 6-well plates and grown to confluence. Wounds were created and photomicrographs were taken immediately and at 24 h after wounding. The width of the cell-free area was measured, and the percentage of migration was considered as the ratio of the reduction of width to the initial width of scraped area (0 h).

### Transwell migration and invasion assay

Transwell chambers were used for cell migration and invasion assays with or without a Matrigel (BD Biosciences) coating. Briefly, serum-free medium was added to the upper chamber (Corning, USA) and cells were cultured in the upper chamber. While medium with 10% FBS was added to the lower chamber. After incubation for 24-36 hours, cells that migrated or invaded through the membrane were stained and counted.

### Flow cytometry

Cell cycle and cell apoptosis analysis were performed by flow cytometry. Briefly, after harvested, cells were fixed overnight with ice-cold 70% ethanol. Then cells were washed and stained for 30 min at room temperature in the dark with 0.5ml of propidium iodide and RNase (BD), and analyzed using a flow cytometer (FACSCalibur, BD). For cell apoptosis analysis, cells were harvested and stained with Annexin V and propidium iodide (BD).

### Xenograft assays in nude mice

Animal use and experiment protocol were approved by the Animal Ethics Committee of Central South University. A2bR-shRNA T24 cells (5×10^6^) were inoculated subcutaneously into the right back of 6-week-old BABL/c nude mice (*n* = 5 for each group). Tumor volume was measured twice a week since 7 days after implantation, and calculated according to the formula: tumor volume (mm^3^)=(width^2^×length)/2. On day 35th, the tumor tissues were removed.

### Statistical analysis

The statistical analysis was conducted by using SPSS 14.0 software (SPSS Inc., Chicago, IL, USA). The data are expressed as the mean ± SD. The categorical variables were analyzed by using Pearson's chisquare test or Fisher's exact probability test. The Kaplan-Meier test was performed for survival analysis. Multivariate analysis was determined by using the Cox proportional hazards model. *P* value < 0.05 was considered statistically significant.

## SUPPLEMENTARY MATERIAL FIGURE AND TABLE



## References

[1] Siegel RL, Miller KD, Jemal A (2015). Cancer statistics, 2015. CA Cancer J Clin.

[2] Kaufman DS, Shipley WU, Feldman AS (2009). Bladder cancer. Lancet.

[3] Parekh DJ, Bochner BH, Dalbagni G (2006). Superficial and muscle-invasive bladder cancer: principles of management for outcomes assessments. J Clin Oncol.

[4] Cookson MS (2005). The surgical management of muscle invasive bladder cancer: a contemporary review. Semin Radiat Oncol.

[5] Dai Y, Zhang W, Wen J, Zhang Y, Kellems RE, Xia Y (2011). A2B adenosine receptor-mediated induction of IL-6 promotes CKD. J Am Soc Nephrol.

[6] Tang J, Jiang X, Zhou Y, Xia B, Dai Y (2015). Increased adenosine levels contribute to ischemic kidney fibrosis in the unilateral ureteral obstruction model. Exp Ther Med.

[7] Tang J, Jiang X, Zhou Y, Dai Y (2015). Effects of A2BR on the biological behavior of mouse renal fibroblasts during hypoxia. Mol Med Rep.

[8] Hasko G, Linden J, Cronstein B, Pacher P (2008). Adenosine receptors: therapeutic aspects for inflammatory and immune diseases. Nat Rev Drug Discov.

[9] Mousavi S, Panjehpour M, Izadpanahi MH, Aghaei M (2015). Expression of adenosine receptor subclasses in malignant and adjacent normal human prostate tissues. Prostate.

[10] Kasama H, Sakamoto Y, Kasamatsu A, Okamoto A, Koyama T, Minakawa Y, Ogawara K, Yokoe H, Shiiba M, Tanzawa H, Uzawa K (2015). Adenosine A2b receptor promotes progression of human oral cancer. BMC Cancer.

[11] Cekic C, Sag D, Li Y, Theodorescu D, Strieter RM, Linden J (2012). Adenosine A2B receptor blockade slows growth of bladder and breast tumors. J Immunol.

[12] Fredholm BB, Irenius E, Kull B, Schulte G (2001). Comparison of the potency of adenosine as an agonist at human adenosine receptors expressed in Chinese hamster ovary cells. Biochem Pharmacol.

[13] Hajiahmadi S, Panjehpour M, Aghaei M, Mousavi S (2015). Molecular expression of adenosine receptors in OVCAR-3, Caov-4 and SKOV-3 human ovarian cancer cell lines. Res Pharm Sci.

[14] Wojnarowicz PM, Breznan A, Arcand SL, Filali-Mouhim A, Provencher DM, Mes-Masson AM, Tonin PN (2008). Construction of a chromosome 17 transcriptome in serous ovarian cancer identifies differentially expressed genes. Int J Gynecol Cancer.

[15] Li S, Huang S, Peng SB (2005). Overexpression of G protein-coupled receptors in cancer cells: involvement in tumor progression. Int J Oncol.

[16] Xiang HJ, Liu ZC, Wang DS, Chen Y, Yang YL, Dou KF (2006). Adenosine A(2b) receptor is highly expressed in human hepatocellular carcinoma. Hepatol Res.

[17] Ma DF, Kondo T, Nakazawa T, Niu DF, Mochizuki K, Kawasaki T, Yamane T, Katoh R (2010). Hypoxia-inducible adenosine A2B receptor modulates proliferation of colon carcinoma cells. Hum Pathol.

[18] Mittal D, Sinha D, Barkauskas D, Young A, Kalimutho M, Stannard K, Caramia F, Haibe-Kains B, Stagg J, Khanna KK, Loi S, Smyth MJ (2016). Adenosine 2B Receptor Expression on Cancer Cells Promotes Metastasis. Cancer Res.

[19] Ihara T, Hosokawa Y, Kumazawa K, Ishikawa K, Fujimoto J, Yamamoto M, Muramkami T, Goshima N, Ito E, Watanabe S, Semba K (2016). An in vivo screening system to identify tumorigenic genes. Oncogene.

[20] Schulte G, Fredholm BB (2003). The G(s)-coupled adenosine A(2B) receptor recruits divergent pathways to regulate ERK1/2 and p38. Exp Cell Res.

[21] Linden J, Thai T, Figler H, Jin X, Robeva AS (1999). Characterization of human A(2B) adenosine receptors: radioligand binding, western blotting, and coupling to G(q) in human embryonic kidney 293 cells and HMC-1 mast cells. Mol Pharmacol.

[22] Wei Q, Costanzi S, Balasubramanian R, Gao ZG, Jacobson KA (2013). A2B adenosine receptor blockade inhibits growth of prostate cancer cells. Purinergic Signal.

[23] Fernandez-Gallardo M, Gonzalez-Ramirez R, Sandoval A, Felix R (2016). Adenosine Stimulate Proliferation and Migration in Triple Negative Breast Cancer Cells. PLoS One.

[24] Desmet CJ, Gallenne T, Prieur A, Reyal F, Visser NL, Wittner BS, Smit MA, Geiger TR, Laoukili J, Iskit S, Rodenko B, Zwart W, Evers B (2013). Identification of a pharmacologically tractable Fra-1/ADORA2B axis promoting breast cancer metastasis. Proc Natl Acad Sci U S A.

[25] Kim HC, Kim YS, Oh HW, Kim K, Oh SS, Kim JT, Kim BY, Lee SJ, Choe YK, Kim DH, Kim SH, Chae SW, Kim KD (2014). Collagen triple helix repeat containing 1 (CTHRC1) acts via ERK-dependent induction of MMP9 to promote invasion of colorectal cancer cells. Oncotarget.

[26] Yang J, Lv X, Chen J, Xie C, Xia W, Jiang C, Zeng T, Ye Y, Ke L, Yu Y, Liang H, Guan XY, Guo X (2016). CCL2-CCR2 axis promotes metastasis of nasopharyngeal carcinoma by activating ERK1/2-MMP2/9 pathway. Oncotarget.

[27] Godde NJ, Sheridan JM, Smith LK, Pearson HB, Britt KL, Galea RC, Yates LL, Visvader JE, Humbert PO (2014). Scribble modulates the MAPK/Fra1 pathway to disrupt luminal and ductal integrity and suppress tumour formation in the mammary gland. PLoS Genet.

[28] Merighi S, Simioni C, Gessi S, Varani K, Mirandola P, Tabrizi MA, Baraldi PG, Borea PA (2009). A(2B) and A(3) adenosine receptors modulate vascular endothelial growth factor and interleukin-8 expression in human melanoma cells treated with etoposide and doxorubicin. Neoplasia.

