# Supplementary material for: The adenosine A2b receptor promotes tumor progression of bladder urothelial carcinoma by enhancing MAPK signaling pathway
Source: Oncotarget. 2017 May 12;8(30):48755–68. doi: 10.18632/oncotarget.17835 (PMC5564722; doi:10.18632/oncotarget.17835)
Supplement: Supplementary file 1 [file oncotarget-08-48755-s001.pdf]

The adenosine A2b receptor promotes tumor progression of bladder urothelial carcinoma by enhancing MAPK signaling pathway

Supplementary Material

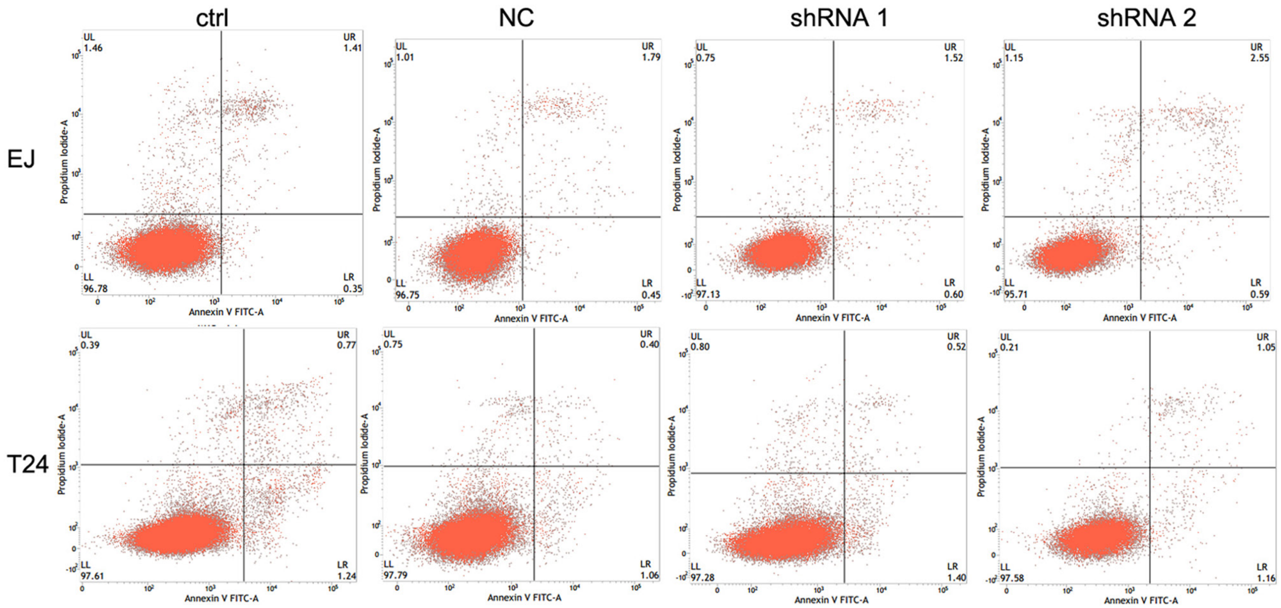

Supplementary Figure S1: Flow cytometry analysis shows that knockdown of A2bR expression does not affect cell apoptosis

Supplementary Table S1: Primers were used for Real-Time PCR

Gene	Forward primer	Reverse primer
A1R	5′- AACAATCTGAGTGCGGTGGAG -3′	5′- TGCTTGCGGATTAGGTAGAAGAC -3′
A2aR	5′- AAAGGAGGGCAAGAACCACT -3′	5′- ACCAGCACACAGGCAAAGA -3′
A2bR	5′- ATCCCATTGTCTATGCTTACCG -3′	5′- CATTCCCACTCTTGACATCTGC -3′
A3R	5′- CCTGGCTGACATTGCTGTT -3′	5′- TGGAGGCGTGGGTAAAGATA -3′
β-actin	5′- TGACGTGGACATCCGCAAAG -3′	5′- CTGGAAGGTGGACAGCGAGG -3′
